# Follicular Lymphoma Microenvironment: An Intricate Network Ready for Therapeutic Intervention

**DOI:** 10.3390/cancers13040641

**Published:** 2021-02-05

**Authors:** Cèlia Dobaño-López, Ferran Araujo-Ayala, Neus Serrat, Juan G. Valero, Patricia Pérez-Galán

**Affiliations:** 1 Department of Hematology-Oncology, Institut d’Investigacions Biomèdiques August Pi I Sunyer (IDIBAPS), 08036 Barcelona, Spain; cdobanol@clinic.cat (C.D.-L.); faraujo@clinic.cat (F.A.-A.); mnserrat@clinic.cat (N.S.); garcia32@clinic.cat (J.G.V.); 2 Centro de Investigación Biomédica en Red-Oncología (CIBERONC), 28029 Madrid, Spain

**Keywords:** follicular lymphoma, microenvironment, immunotherapy

## Abstract

**Simple Summary:**

Follicular lymphoma is a paradigm of tumors that require the interaction between tumor and microenvironment cells to foster their development from initial steps to progression. Recent large-scale genome studies have uncovered multiple genetic alterations that cooperate with the lymphoma microenvironment to promote cell survival, proliferation and to facilitate tumor evasion from host immune system. Understanding the crosstalk between tumor B-cells and the microenvironment is fundamental to identify vulnerabilities that may offer novel therapeutic targets. This review highlights recent findings showing the effect of common genetic mutations modulating the cell composition and phenotype of the tumor microenvironment and the novel therapeutic perspectives to target these interactions.

**Abstract:**

Follicular Lymphoma (FL), the most common indolent non-Hodgkin’s B cell lymphoma, is a paradigm of the immune microenvironment’s contribution to disease onset, progression, and heterogeneity. Over the last few years, state-of-the-art technologies, including whole-exome sequencing, single-cell RNA sequencing, and mass cytometry, have precisely dissected the specific cellular phenotypes present in the FL microenvironment network and their role in the disease. In this already complex picture, the presence of recurring mutations, including *KMT2D, CREBBP, EZH2,* and *TNFRSF14*, have a prominent contributory role, with some of them finely tuning this exquisite dependence of FL on its microenvironment. This precise characterization of the enemy (FL) and its allies (microenvironment) has paved the way for the development of novel therapies aimed at dismantling this contact network, weakening tumor cell support, and reactivating the host’s immune response against the tumor. In this review, we will describe the main microenvironment actors, together with the current and future therapeutic approaches targeting them.

## 1. FL Microenvironment: Friend or Foe?

Follicular lymphoma (FL), the most common indolent non-Hodgkin’s lymphoma (NHL), is a biologically heterogeneous disease with clinical variations in patient outcome [1].

The initial oncogenic hit happens in the Pre B/Pro B stage of B cells in the bone marrow (BM) where they acquire the t (14;18) translocation due to an error in V(D)J recombination. Subsequently, these cells home to B cell follicles inside lymph nodes (LNs) where they encounter the antigen (Ag) and undergo, in the germinal center (GC), somatic hypermutation (SHM) and class switch recombination (CSR) (IgM to IgG) of the immunoglobulin that constitutes the B cell receptor (BCR). FL-like cells interact with follicular dendritic cells (FDCs) and are selected to undergo apoptosis or be rescued by follicular helper T cells (T_FH_), based on the Ag affinity of their BCRs. Overexpression of BCL2, along with additional anti-apoptotic proteins, allow apoptosis escape independently of BCR affinity. These FL-like B cells then exit the GC and enter circulation where they might be prone to traffic between secondary lymphoid organs and the BM, and they acquire additional genetic changes necessary for transformation to FL, such as *CREBBP*, *KMT2D*, *EZH2*, *TNFRS14*, among others [2,3]. 

There is now growing evidence that crosstalk between lymphoma cells and stromal and immune cells in lymphoid compartments is fundamental for disease onset and progression. This crosstalk is dynamic and shapes the tumor microenvironment enhancing the pro-tumoral features of the niche [4,5]. FL represents a paradigm of dependence on the microenvironment. Seminal microarray studies in LN biopsies from the Leukemia and Lymphoma Profiling Project (LLMPP) series established for the first time that FL prognosis was not given by the tumor cell per se but by the composition of non-malignant cells [6,7,8]. Subsequently, many studies have tried to identify phenotypic markers to stratify patients, although this picture has been more complicated than anticipated, and influenced by treatment.

**Figure 1:**
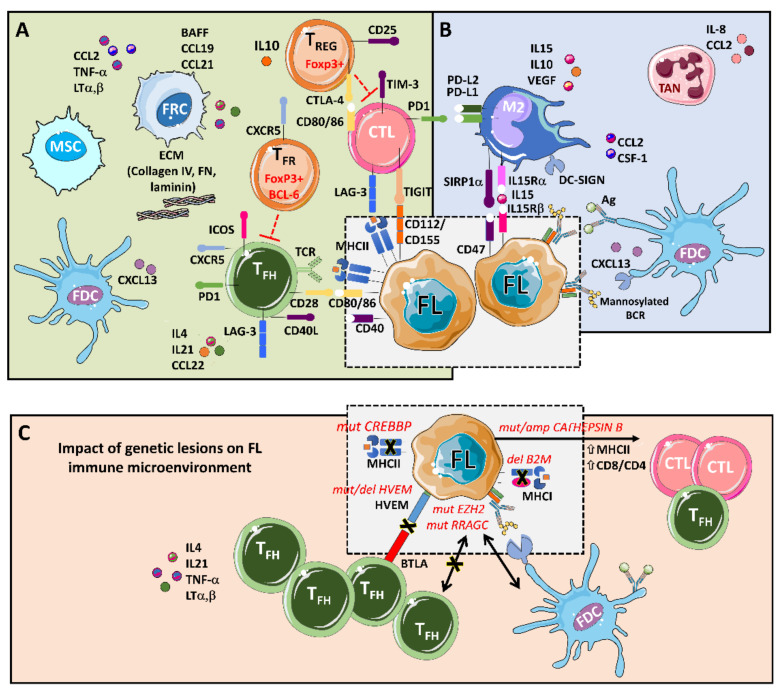
An integrative view of follicular lymphoma (FL) microenvironment and its crosstalk with genetic drivers. (**A**) FL is highly infiltrated with several T cell subpopulations, where T_FH_ are fundamental players through MHC II and CD40L, while immunosuppressive T_REG_ hamper cytotoxic T cells (CTLs) activation. Fibroblastic reticular cells (FRCs) also participate in immunosuppression by secreting extracellular matrix (ECM) proteins that regulate T cell trafficking and cooperate with T_FH_. (**B**) FL cells favor the recruitment of monocytes through CCL2 and CSF-1 that differentiate and polarize mostly into M2-like macrophages expressing PD-L1 and PD-L2 and dampening CTLs cytotoxic activity. Both macrophages and FDCs activate B cell receptors (BCRs) through lectins binding to mannosylated BCR. Likewise, FDCs also activate BCRs through the presentation of immunocomplexes to FL cells. Neutrophils are recruited through IL8 secretion in the FL niche and support lymphoma growth. (**C**) Several genetic alterations corrupt the microenvironment to better support FL. Mutations in *CREBBP* and deletions in *B2M* genes reduce MHC II and MHC I expression, respectively. On the contrary, aberrant *CATHEPSIN B* leads to an increase in MHC II expression and CD8 expansion. Both *EZH2* and *RRAGC* mutations reduce the need for T_FH_ help making FL cells more dependent on FDCs, while disruption of the HVEM–BTLA axis allows uncontrolled T_FH_ support to FL cells.

The main players that support tumors, through a complex set of cytokines, receptors, immune modulators, and pro-angiogenic factors, are follicular dendritic cells (FDCs), fibroblastic reticular cells (FRCs), mesenchymal stromal cells (MSCs), and tumor-associated macrophages (TMAs), together with a rich T cell infiltrate composed of CD4 T follicular helpers (T_FH_) cells, CD4 T follicular regulatory (T_FR_) cells, CD4 T regulatory cells (T_REG_), and CD8 cytotoxic T cells (CTL) [9,10] (Figure 1).

The FL–LN maintains a structure reminiscent of a normal LN, where B cells are supported by T_FH_ and the follicles are delimitated for a network of FDCs. These types of dendritic cells are only present in the follicles of primary and secondary lymph organs. FDCs are particular Ag-presenting cells (APCs) as they do not internalize, process, and present Ag, but present intact Ag–Ab complexes on their cell surface that induce survival of FL cells and their differentiation into memory B cells or plasma cells. In vitro studies using a non-immortalized FDC cell line have demonstrated that FDCs preferentially bind to GC B cells and deliver a positive signal for B cell survival, activation, and differentiation [11,12]. Interestingly, FL cells are then able to present this Ag derived from FDC presentation and trigger T_FH_ recruitment. T_FH_ are specialized CD4^+^ T cells located in the GC light zone and are characterized by CD4^+^, CXCR5^+^, PD1^+^, ICOS^+^, and CD25^-^ phenotype. T_FH_ are essential for the formation and maintenance of GC, contributing to B cell fitness by means of CD40L signaling and IL-4 or IL-21 cytokines [13]. It is noteworthy that IL-4 proteins are five-fold more abundant in FL germinal centers than in normal tonsil [14]. Moreover, malignant B cells are involved in the recruitment of T_REG_ (CD4^+^, CD25^+^), present in a higher frequency in FL compared with tonsils, and acting as inhibitors of CD8^+^ T cell effector activity [15].

FRCs are stromal cells present in the T cell zone of the LN that are endowed with functions that create a permissive niche by secreting components of the extracellular matrix (ECM), including laminin, fibronectin, and collagen IV. They organize and regulate immune cell trafficking, differentiation, and migration of T cells through an IL-4/CXCL12 communication axis with T_FH_, among other signals, and secretion of additional chemokines such as CCL19 and CCL21. FRC also plays a direct role in B malignant cells’ activation and survival through BAFF signaling [16,17,18,19]. 

MSCs are present both in the BM niche and LNs, supporting B cell survival through the secretion of numerous factors, such as BAFF, TNFα, lymphotoxin α (LTα) [20], while chemokine CCL2 favors the recruitment of macrophages to the FL niche [21].

Tumor-associated macrophages (TAMs) are highly plastic cells from the myeloid lineage. Depending on the stimuli, macrophages can be polarized into M1 (inflammatory phenotype) or M2 (anti-inflammatory), resulting in distinct cytokine production or T cell function (Th1 and Th2). We have recently demonstrated that FL-FDC niche promotes, via the secretion of CCL2 and CSF-1, monocyte recruitment, differentiation, and polarization towards an M2-like pro-tumoral phenotype, as seen in FL patient biopsies [5], favoring angiogenesis, dissemination, and immunosuppression [22]. Moreover, macrophages express C-type lectin dendritic cell–specific intercellular adhesion molecule-3-grabbing non-integrin (DC-SIGN) that binds to mannosylated BCR, activating B cell survival independently of Ag [23,24,25]. In addition, monocyte/macrophages trans-present IL-15 to B cells in the FL niche and cooperate with T-cell-derived CD40L to promote IL-15-dependent B-cell proliferation [26].

Thus, FL is surrounded by a rich and well-interconnected network of supportive allies that may account for the incurability of this indolent lymphoma. Moreover, this dynamic microenvironment also takes part in the histological transformation (HT) of FL to an aggressive lymphoma. These modifications comprise the disruption of the FDC network [27], changes in the gene expression of CD4/8 T cells leading to decrease motility [28], a decrease in the number and follicular distribution of FoxP3^+^ T_REG_ [29] and PD-1 positive T cells [30].

## 2. FL Mutational Landscape and Microenvironment Interplay

Although t(14;18) (q32;q21) was discovered decades ago [31], it is considered the first oncogenic hit in FL. It is not present in approximately 10% of patients [32,33], and it has been detected in some healthy individuals [34,35]. Therefore, additional mutations must contribute to disease onset. In this genomic era, the FL genome has been fully characterized by whole genome and exome sequencing and, more recently, by single-cell RNA sequencing (sc-RNAseq). In this review, we focus on those mutations with an impact on the FL microenvironment remodeling.

FL is a malignancy addicted to epigenetic mutations [36] where these hits (*KTMD2, CREBBP/EP300,* and *EZH2* genes) constitute early oncogenic events present in virtually all FL patients [37,38,39]. These genes are involved in the post-translational modification of histones [40]. The loss of function of histone K3K4 methyltransferase KMT2D (also known as MLL2) is the most frequent alteration (60–90%), followed by loss of function mutations in the H3K27/H3K18 acetyltransferases CREBBP (50–70%) and EP300 (10–20%), resulting in transcriptional repression [41,42].

Histone-lysine N-methyltransferase 2D (KMT2D) function is related to GC formation and CSR, two critical steps in the maturation process of B cells, and it cooperates with BCL-2 in lymphomagenesis [41,43]. No impact on the immune microenvironment has been described thus far.

CREB-binding protein (CREBBP) is a haploinsufficient tumor suppressor that acts as a major regulator of enhancer networks in the GC, especially in the light zone, and avoids terminal B cell differentiation [42]. CREBBP also acetylates non-histone proteins such as p53 and BCL6. Loss of function CREBBP mutations leads to reduced activation of p53 as well as to diminished inactivation of BCL6 [44,45]. Noteworthy, *CREBBP* mutations have a clear effect on the immune microenvironment. MHC class II is reduced in FL cells at both the transcriptome and protein levels, resulting in a diminished Ag presentation [46]. Recently, this finding has been confirmed by scRNA-seq analysis [47]. Furthermore, *CREBBP* mutations are associated with reduced T cell proliferation and have been identified as early mutations, since they are present in the earliest inferable progenitors [46] and could even be present in hematopoietic stem and progenitor cell compartments [45]. Altogether, there is a large amount of evidence indicating that *CREBBP* mutations may play a major role in the evasion of immune surveillance during the development of FL tumors. In addition, both *mutCREBBP* and *mutEP300* contribute to lymphomagenesis by enabling unopposed suppression of enhancers by BCL6/SMRT/HDAC3 complexes, suggesting HDAC3-targeted therapy as a precision approach for CREBBP-mutant lymphomas [48], and recent results with specific HDAC3 inhibitors have demonstrated the reactivation of immune responses [49]. While specific HDAC3 inhibitors are not at a clinical stage, diminished chromatin acetylation by *mutCREBBP* might be reverted using pan-HDACi. Preclinical data suggest that these families of compounds may be beneficial in combination with immunotherapy in B cell lymphomas [50]. Some clinical trials have explored pan-HDAC in monotherapy. Vorinostat yielded moderate responses (<50%) in two phase II clinical trials [51,52], while abexinostat has shown an improved overall response rate (ORR) [53] (Table 1). The lack of isoform-specificity could lead to immunosuppressive effects by pan-HDACi [54]. 

*Enhancer of zeste homolog 2* (EZH2) is a histone methyltransferase that methylates H3K27 and presents monoallelic gain-of-function mutations at three recurrent hotspots (Y646, A682, and A692) in 20–30% of FL [73]. It is the combined function of wt and *mutEZH2* that causes an increased di- and trimethylation of H3K27 [74]. EZH2 plays a central role during GC formation in cooperation with BCL6 through the formation of bivalent promoters [75,76]. BCL6 is necessary to maintain GC reaction and acts as a transcriptional repressor, antagonizing CREBBP/EP300 function [42]. Noteworthy is EZH2′s participation also in the remodeling of the GC immune microenvironment. *mutEZH2* cells in the GC light zone lose dependence on T_FH_ cells, while upregulated genes are involved in an interaction with FDC and become dependent on them. As a consequence, these cells are no longer capable of reentering the dark zone of GC but can proliferate as centrocytes [77].

As EZH2 mutations gain function (in contrast to CREBBP/EP300 and KMT2D), they are easily druggable, and several attempts have been made to develop selective inhibitors [78,79]. Nowadays, tazemetostat is the most promising compound (Table 1). FL patients with *mutEZH2* had an objective response of 63% and 71%, higher than in *wtEZH2* patients (28% and 33%, respectively) [80]. These results were confirmed in another phase II study involving 99 patients with relapsed or refractory (R/R) FL, separated in a EZH2^mut^ cohort with an ORR of 69% and progression-free survival (PFS) of 13.8 months and a EZH2WT cohort presenting a diminished ORR of 35% and PFS of 11.0 months [55], which led to FDA approval in June 2020 for adult patients with R/R FL with EZH2 mutated tumors.

B2-microglobulin (B2M) and CD58 genes are inactivated in some DLBCL cases, uncovering a mechanism of immune evasion, as B2M is involved in the expression of MHC class I, necessary for cytotoxic CD8+ T cell recognition and CD58 mediates T and NK cell response [81]. Although initially, no mutations were observed in FL, later studies have shown that mutations and deletions in B2M and CD58 were present in transformed FL [82,83].

Cathepsin S (CTSS), which is aberrant in approximately 20% of FL patients by activating point mutation or amplification [84], has effects opposite to *CREBBP* mutations regarding Ag presentation since it is required for the binding of MHC to antigenic peptides [85]. When cathepsin S is hyperactivated, it cleaves its substrates more efficiently, including CD74, leading to upregulation of MHC class II genes and a higher CD4^+^ T cell infiltration [84,86]. Furthermore, patients carrying CTSS mutations expressed higher levels of IFN-γ and IFN-γR1 [84]. Moreover, while CREBBP mutations have been related to bad prognosis [39], cathepsin S activation correlates with a better outcome after chemoimmunotherapy treatment [84]. It could be explained due to the enhanced CD4^+^ T cell response. In addition, in a CTSS KO in vivo model, the modification of the Ag repertoire supported a multiclonal expansion of cytotoxic CD8+ T cells, and in FL patients, there was an inverse correlation between CTSS and PD1 expression [86].

Herpesvirus entry mediator A (HVEM), also known as TNFRSF14, is a receptor inactivated by mutations and/or deletions in half of FL patients and associated generally with a bad prognosis [87], although some controversy still remains [88].

This loss of function disrupts the interaction between HVEM and BTLA that normally provides inhibitory signals to BCR signaling. In addition, BTLA does not present mutations, but it is often transcriptionally silenced. The net balance is an increased BCR stimulation. Furthermore, HVEM loss makes TME more tumor-supportive due to the high secretion of stromal activating cytokines (TNFα, LTα, LTβ) and increased T_FH_ cell recruitment [67]. A therapeutic approach has been proposed to restore HVEM expression using an engineered chimeric antigen receptor T cell (CAR-T) construct directed towards B cell marker CD19, which produces HVEM protein locally and continuously, and promising results have been shown in mouse lymphoma models [67].

Ras-related GTP binding C (RRAGC) activating mutations occur in up to 17% of patients and constitute a mechanism that bypasses amino acid deprivation to activate mTORC1 signaling [89,90]. As recently published by Ortega-Molina et al., engineered mice are able to activate mTORC1 via PI3K-Akt by ligands that mimic paracrine T cell-derived activating signals (IL-4/CD40L axis). Furthermore, mutated RRAGC confers enhanced B cell activation but in an autonomous manner, reducing T_FH_ dependence, similar to EZH2 mutation. Intriguingly, RRAGC mutation confers an opportunity to target mTORC1 in selected patients by rapamycin [91]. In recent years, several phase I and II clinical trials have been engaged to evaluate the use of temsirolimus or everolimus as single agents [56] and in combination with other drugs. Among these combinations, we highlight temsirolimus with bendamustine and rituximab, achieving an ORR of 90% [57] and with the proteasome inhibitor, bortezomib, resulting in an ORR of 56% [58] (Table 1).

## 3. T Cells: Fundamental Actors in FL Pathogenesis Modulated with BCR Inhibitors

As described above, FL-infiltrated lymphoid tissues preserve normal follicle architecture in the GC, where FL cells behave like normal B cells, interacting with T cells through the MHC class II and responding to activating signals [92]. Accordingly, FL cells remain dependent on BCR signaling, which has made BCR inhibitors [93] (mainly BTK and PI3K inhibitors) useful drugs for treatment. While BCR stimulation can be achieved by ways other than cognate B–T interaction, such as DC-SIGN binding to mannosylated residues of BCR [23], FL still requires cell-to-cell interaction with T_FH_ for proliferation and survival [84,85] (Figure 1). Numerous pieces of evidence indicate that FL cells take advantage of T_FH_ function in order to obtain positive signals that fuel tumor growth and actively modify GC composition by recruiting cells or altering their phenotype in a process called microenvironment re-education [86,87]. Another important actor in the FL milieu is the chemokine CCL22, secreted by FL tumor cells as a result of the CD40L–CD40 axis activated by T_FH_. CCL22 facilitates the active recruitment of T_REG_ and IL-4-producing T cells, which, in turn, may stimulate more chemokine production in a feed-forward cycle [15,94].

The role of T_REG_ FOXP3+ cells in FL prognosis has remained controversial for a long time [29,95]. The discovery of a new T subset called follicular regulatory T cells (T_FR_), which are present in the germinal center and suppress T_FH_ and B cell activation [96], has finally explained the divergence observed in the results. The FOXP3+ population encompasses two different T cell subsets. Conventional T_REG_ can be attracted to the GC through CCL22 secretion, impairing T CD4+ or CD8+ activation by tumor antigens, with a pro-tumoral consequence. On the contrary, the T_FR_ population probably plays an antitumoral role, as they reduce T_FH_ support to malignant cells. 

It is noteworthy that PI3Kδ inhibitors have been shown to disrupt this interactive B–T cell network, blocking this forward-feed cycle efficiently. Idelalisib has been the first-in-class PI3Kδ inhibitor FDA-approved in 2014 for the treatment of relapsed or refractory (R/R) FL. In a phase 1b study for indolent NHL, 47% of patients showed ORR, with one patient demonstrating a complete response [97,98]. Further trials in FL confirmed these values (57% ORR, 6% CR) [59], demonstrating that PI3Kδ inhibition has significant efficacy in FL, although it also has serious side effects, mainly severe diarrhea, hepatotoxicity, or pneumonitis, and, in some cases, made it necessary to stop the treatment [99].PI3Kδ is a key regulator of T_FH_ differentiation [100] and has deleterious effects on T_REG_ [101], which closely relates with some side effects described for this drug and may be responsible for its therapeutic activity. In this regard, using ex vivo FL-FDC primary co-cultures, we recently uncovered that idelalisib interferes with the CD40/CD40L pathway at the B–T interface, decreasing CD40L-induced proliferation and downregulating the expression of key membrane proteins critical for B–T cell synapses (CD80, SLAMF1, and ICAM1). The net balance of these effects might result in inefficient crosstalk between FL cells and the supportive T_FH_ cells. Moreover, the chemokine CCL22, fundamental in the FL milieu, decreases after idelalisib treatment, and this phenomenon impacts on the composition of FL microenvironment by a decrease in the recruitment of T_REG_ and T_FH_, but not T_FR_ into FL-FDC niche, which may allow the host to mount superior immune responses against the tumor [60].

In the last few years, new PI3K inhibitors were added to the list: copanlisib that inhibits all class I PI3Ks, including α isoform and duvelisib, a dual PI3K γ, δ inhibitor. In a phase II study, copanlisib ORR was 43.7% in indolent lymphomas [93]. With respect to undesirable effects, copanlisib produces less severe diarrhea or hepatotoxicity than PI3Kδ inhibitor, but it displays new side effects like hyperglycemia or hypertension, all of them generally manageable. On the basis of its improved safety, there are currently three phase III trials on copanlisib in indolent lymphoma [61]. The advantages of duvelisib are related to γ isoform inhibition, which has a special effect on myeloid cells [102] (Section 5.2.). Finally, combinations of PI3K inhibitors with different agents are under investigation (Table 1). 

In addition to PI3K inhibitors, additional BCR inhibitors have been developed and tested in FL. 

The BTK inhibitor, ibrutinib, was tested as monotherapy in FL, with modest results (ORR 37.5%) [63]. However, its combination with a Rituximab in a randomized phase III trial has yielded encouraging results in untreated FL patients [64]. In chronic lymphocytic leukemia (CLL), it has been demonstrated that ibrutinib profoundly reshapes the T cell compartment, improving T cell function. Ibrutinib induces expansion of memory T cells, Th1 polarization, reduces the expression of inhibitory receptors (i.e., PD-1 and CTL-4), and improves immune synapse between T cells and CLL cells [103,104]. However, no data are available for FL in this regard. Finally, the SYK/JAK inhibitor cerdulatinib has shown significant tumor responses in refractory B cell lymphoma [105].

Overall, these kinase inhibitors impact not only on BCR signaling but also on other receptors such as CD19, CD40, or IL-4R. In consequence, these drugs can cause indirect inhibition of T_FH_ supportive action. Additionally, they have a direct effect on T cells and macrophages that will contribute to antitumor activity and often to its undesirable effects.

## 4. Immune Escape in FL and Checkpoint Inhibitors

Immune escape is a hallmark of cancer and FL is not an exception. FL cells have evolved to avoid immune surveillance via multiple mechanisms, including advantageous somatic mutations (described in Section 2), but also through modulation of specific genes to inactivate both innate and cellular immunity.

As described in the previous section, the FL microenvironment is characterized by a heavy infiltration of T cells. However, early functional studies in FL samples determined that tumor-infiltrating T cells (TILs) were not responsive to cytokines such as IL-4, IL-10, or IL-21, in contrast to those of peripheral blood (PB) from the same patients, suggesting that immune-suppressive molecules may be present in the tumor tissue [106]. T cell activity is regulated by immune checkpoint activators (CD40L, OX40, CD27, CD28, and 4-1BB/CD137) and inhibitors (CTLA-4, PD1, LAG-3, TIM-3, and TIGIT) [107]. We briefly described the immune checkpoint inhibitors that may be relevant in FL pathogenesis. 

Programmed death 1 (PD-1) is upregulated in a large proportion of tumor-infiltrating lymphocytes (TILs) in many different tumor types, but it is also upregulated in other immune cells. Intracellular PD-1 signaling is activated upon PD-1 binding to its ligands PD-L1 (B7-H1, CD274), or PDL2 (PDCD1LG2, CD273), which induces a reduction in the T cell activation cascade. Thus, by expressing PD-1 ligands on the tumor cell surface and engaging PD-1-positive infiltrating lymphocytes, tumors utilizing the PD-1 pathway can therefore evade an immune response. 

Cytotoxic T-lymphocyte-associated protein 4 (CTLA-4/CD152) is a homolog of CD28 and binds to CD80/CD86. CTLA-4 is constitutively expressed in T_REG_ and activated T cells.

Lymphocyte activation gene 3 (LAG3/CD223), a CD4-like molecule, is upregulated on activated CD4+ and CD8+ T cells and a subset of natural killer (NK) cells upon binding to MHC class II molecules, inducing the inhibition of T lymphocyte activity and eventually its anergy [108].

T cell immunoglobulin-3 (TIM-3) is expressed in T_FH_, CTLs and NKs, and is co-regulated and co-expressed along with PD-1, LAG-3, or TIGIT on CD4^+^ and CD8^+^ T cells, and it marks the most dysfunctional or terminally exhausted subset of CD8^+^ T cells [109].

T cell immunoglobulin and ITIM domain (TIGIT) shifts the cytokine balance by targeting the immune response at multiple levels, namely, through its action on APCs, CTLs, and T_REG_ cells. In DCs, TIGIT ligation induces IL-10 production and dampens type 1 immunity indirectly. 

In FL, PD1 is expressed in both intratumoral CD4 and CD8 T cells, but several subsets with different expression levels have been identified [110]. CD4^+^PD-1^high^ T cells predominantly reside in the LN follicles, while PD-1^low^ T cells are mainly located in interfollicular areas. CD8 T cells are mainly PD1^low^, and a significant portion express TIM-3. Intratumoral CD4^+^PD-1^high^ T cells have a T_FH_ cell phenotype that supports tumor growth with no TIM-3 expression, while CD4^+^PD-1^low^ T cells that have an exhausted phenotype, express TIM-3 and display a reduced cytokine production and cell–signal transduction. Moreover, T cells from the LNs of FL patients present a high percentage of CD8+TIM-3+ showing defective cytokine production upon TCR engagement, despite the presence of ex vivo markers of lytic granule [111].

Regarding PD1 ligands, it is accepted that while FL cells do not express PD-L1 and PD-L2 is moderately expressed in a high proportion of FL cases, these ligands are present in the tumor microenvironment [112]. Precisely, PD-L1^+^ histiocytes have been detected in the T cell-rich zone of the neoplastic follicles [106].

Overall, these studies suggest that in FL, PD-1 expression is not sufficient to distinguish exhausted from activated T cells, and immune escape in FL goes far beyond PD-1. Data mining analysis studies [112] identified genes involved in cancer immune–evasion pathways (immune escape gene set (IEGS)) across FL and normal B cell transcriptomes indicated that the whole IEGS was significantly upregulated in FL samples compared to normal tonsils. These genes include, besides the PD-1 axis genes, additional immune checkpoints (TIGIT and CTLA-4), exhaustion markers (TIM-3, LAG3, Galectin 1 and 3), chemoattractants of immunosuppressive cells (CSF-1, CCL2, and CCL22) M2 macrophages markers (CD206, CD163) and immunosuppressive molecules (IL10, VEGF, IDO1 and 2). Further validation in FL tissue microarray indicated an abundant immune infiltrate expressing PD-L1^+^, PDL2^+^, and LAG3^+^. LAG-3 has been found in intratumoral PD1+ T cells, and they are phenotypically heterogeneous, with a predominant effector memory phenotype. Intratumoral PD-1^+^LAG-3^+^ T cells exhibited a reduced capacity to produce cytokines and granules compared to PD-1^+^LAG-3^-^ T cells. Moreover, LAG-3 expression may be upregulated on CD4+ or CD8+ T cells by IL-12, cytokine enriched in the serum of FL patients. Furthermore, simultaneous blockade of both PD-1 and LAG-3 signaling enhances the function of intratumoral CD8^+^ T cells. The relevance of LAG-3 expression on intratumoral T cells correlated with a poor outcome in FL patients [113].

Armed CTLs (CD3^+^CD8^+^Granzyme B^+^) represent a rich infiltrate in FL interfollicular areas and are associated with better outcomes [114]. However, these cells express higher TIM-3 expression than their counterparts in normal tonsil. As expected, these cells show defective responses to TCR activation, and a high percentage of TIM-3^+^ immune cells in the infiltrate was associated with shortened patient PFS, independently of Granzyme B (GrzB) score, highlighting the relevance of this checkpoint inhibitor in FL and the need of scoring both TIM-3 and Grz B in this disease [111].

TIGIT [115,116] also constitutes a common inhibitory receptor in FL, expressed by the majority of CD8 T effector memory cells, which are commonly co-expressed with exhaustion markers such as PD-1 and CD244. These FL CD8^+^ T cells showed significantly reduced TCR- induced distal signaling (pERK) and reduced production of IFNγ, while TCR proximal signaling did not seem to be affected. Interestingly, the TIGIT ligands CD112 and CD155 are expressed by FDCs. Dysfunctional TCR signaling correlated with TIGIT expression in FL CD8 T cells and could be fully restored upon in vitro culture, supporting that TIGIT blockade is a relevant strategy for improved immunotherapy in FL, possibly in combination with blockade of PD-1.

In terms of clinical development, the PD1 axis has been the most explored. Nevertheless, despite the initial encouraging results in early Phase 1b trials [117], later assessment in larger cohorts have demonstrated limited activity of nivolumab in R/R FL [68]. Noteworthy is the combination of anti-PD1 with standard anti-CD20 immunotherapy that has been more successful. In this regard, a phase II trial combining anti-PD-L1 obtained significant OR (66%) and a high proportion of complete responses with manageable adverse events [69]. Following the experience in melanoma, PD-1 has been combined with CTLA-4. Recent results of the phase 1b have been quite disappointing, as the combination did not provide added benefit other than the single agents [118].

The low responses of the PD-1 blockade may be related to the heterogeneity of PD-1^+^ T cells in FL and the role of PD-1 in restraining T_FH_ cell help to GC B cells. T_FH_ are principal actors in the FL microenvironment. In view of poor clinical responses to anti-PD1, these therapies may unleash PD1 break in T_FH_ and increase helper signals to FL B cells more prominently than the benefit of unleashing existing CD8. This suggests that additional immunotherapy approaches that do not unleash T_FH_ cell helper signals need to be tested in FL.

In this regard, anti-LAG-3 therapy is in phase I trials and based on encouraging preclinical results in MHC II expressing tumors, such as Hodgkin’s Lymphoma; anti-LAG3 therapy in combination with anti-PD1 is also under clinical investigation. Similarly, anti-TIM-3 alone and in combination with anti-PD1 is also under clinical investigation (Table 1).

Although preclinical results with anti-TIGIT may support its clinical investigation [119], no trials in lymphoma are registered at the moment of writing this review. Nevertheless, several studies are running in advanced solid tumors or plasma cell neoplasms as multiple myeloma.

A complementary approach is to target the immune checkpoint activators. A recent paper using the anti-CD137 antibody in combination with rituximab has shown a favorable safety profile and clinical activity in patients with R/R FL. However, the combination did not enhance clinical activity relative to rituximab alone or other current standards of care. It is noteworthy that those FL patients with CR showed increased T cell infiltration and cytotoxic activity in tumors [70]. CD40 constitutes another druggable activator; however, to date trials performed with agonist antibodies against CD40 have provided modest results and may need to be further tested in combination therapy [120] as the ongoing trial with anti-PDL1 (Table 1).

## 5. Maneuvers of Myeloid Companions in FL

Myeloid cells are a major cellular compartment of the immune system composed of a heterogeneous population of cells like mast cells, dendritic cells, monocytes, macrophages, and granulocytes, all of which originate from the bone marrow but mature into subpopulations with diverse and unique properties [121]. Most of these populations are known to be part of the tumor microenvironment in FL. However, in recent years, monocytes, macrophages, and neutrophils have received most of the attention, as they play an important role in disease severity, transformation, clinical outcome, and response to therapy in this disease [122].

### 5.1. Tumor-Associated Monocytes in FL

As described before, TAMs derive from circulating PB monocytes that originated in the BM. These monocytes are recruited to the tumor tissues and then differentiate locally in response to a variety of cytokines, chemokines, and growth factors produced by the stromal and tumor cells in the tumor microenvironment. For instance, in FL, the chemokine CCL2 from MSCs and macrophage colony-stimulating factor were shown to recruit inflammatory monocytes to the tumor site, and then differentiate into TAMs in response to IL-4, IL-10, IL-13, and other cytokines in the tumor microenvironment and promote lymphoma dissemination [21].

Two studies have analyzed the significance of absolute monocyte count (AMC) in FL. The first study with a large cohort of patients described that AMC was associated with inferior overall survival (OS) in FL independently of FLIPI in a multivariate analysis. The AMC may be most helpful when used in conjunction with the FLIPI, as the AMC was able to identify high-risk patients otherwise identified as low-/intermediate-risk by the FLIPI. Conversely, the AMC was able to identify relatively low-risk patients classified as high-risk by the FLIPI [123]. In contrast with these results, in a second study in a cohort of 150 follicular lymphoma patients who received rituximab and cyclo-phosphamide–doxorubicin–vincristine–prednisone regimen (R-CHOP) therapy, PFS did not differ significantly according to the AMC [124].

The lymphocyte-to-monocyte ratio (LMR) constitutes a prognostic factor in different neoplasms, but its potential relevance in FL is not well defined. An initial retrospective cohort study including 88 patients with a histologically proven FL diagnosis demonstrated that LMR played a significant role in predicting PFS; however, the strength of the evidence for OS was weak [125]. More recently, an extensive study analyzed a cohort of 384 FL patients for which the LMR was available at diagnosis. In these series, patients with an LMR ≤ 2.5 had a shorter PFS and inferior OS. Furthermore, low LMR was also an independent risk factor for histological transformation. Likewise, patients with a low LMR had a higher rate of second malignancies. The authors concluded that LMR could be an additional tool to improve the prognostic classification of FL patients in order to avoid toxicities of overtreating low-risk patients and to intensify therapy in patients at a higher risk of early progression or histological transformation [126].

### 5.2. Tumor-Associated Macrophages in FL

Macrophages are conventionally classified into M1 and M2 subtypes according to their polarization status and functional role in the immune system. M2 macrophages, also known as alternatively activated macrophages, exhibit anti-inflammatory features, downregulating expression of their MHC molecules and interleukin IL-12, while expressing high levels of IL-10, MSR1 (CD204), and arginase. M2 macrophages are involved in wound-healing and angiogenesis. This is in contrast to M1 macrophages associated with antitumor responses and production of high levels of pro-inflammatory cytokines, including TNFα, IL-1, IL-6, IL-12, and inducible nitric oxide synthase [127]. TAMs share some similarities with the M2 macrophage subset because they express a series of markers, such as CD163, the Fc fragment of IgG, C-type lectin domains, and heat shock proteins, some of which are commonly expressed in M2 macrophages. Moreover, the acquisition of an M2-like phenotype is also caused by the secretion of tumor-derived cytokines such as IL4, IL10, and IL13. However, findings suggest that this binary polarization model is becoming obsolete, and there exists a whole spectrum of TAM phenotypes that are yet to be discovered and fully characterized [128].

Since early gene expression studies by Dave et al., highlighting the role of macrophages and other immune cells in FL outcomes [8], subsequent immunohistochemical studies have tried to transfer these findings into the clinical laboratory by associating the cellular composition of the microenvironment and its spatial distribution with the progression of the disease. To date, this issue remains a matter of debate.

The first study in the pre-rituximab era analyzed CD68 in a study group that consisted of uniformly staged FL patients treated with BP-VACOP (bleomycin, cisplatin, etoposide, doxorubicin, cyclophosphamide, vincristine, and prednisone) followed by radiation. In this study, high numbers of lymphoma-associated macrophages predicted inferior survival [129]. However, the introduction of rituximab early changed this view, and high TAMs content correlated with longer survival rates after R-CHOP [130]. These results were later confirmed in patients from the FL-2000 trial, a prospective multicenter study conducted by the GELA (Groupe d’Etude des Lymphomes de l’Adulte) where patients were randomly assigned to receive cyclophosphamide, doxorubicin, etoposide, prednisolone, and interferon (CHVP-I) or rituximab plus CHVP-I. The study demonstrated that high numbers of intratumoral macrophages correlated with poor prognosis in the patients treated with chemotherapy without rituximab [131].

Later on, the identification of TAMs was performed more accurately using the M2 marker CD163, a member of the scavenger receptor cysteine-rich family. In 2010, Clear et al. published an analysis restricted to the interfollicular area from TMAs. Importantly, the treatment was variable during the 35-year period under review, and patients were treated according to the current protocol during this period. This work demonstrated the existence of a correlation between the number of CD163^+^ TAMs in angiogenic sprouts (CD31^+^) with poor prognosis [132]. More recently, an enlightening study investigated the correlation of TAMs with outcome using automated image analysis, analyzing a single-institution experience with uniform therapy (BCCA cohort, R-CVP) in a cohort of 186 patients and compared the findings with those from a prospective, randomized phase III clinical trial (PRIMA, R-CHOP followed by R maintenance) containing 395 samples. This study showed that most FL samples were infiltrated by few macrophages, and increased staining for CD163 was associated with poor PFS and OS in the BCCA cohort and favorable PFS in the PRIMA cohort. On the other hand, the CD68 staining cells did not predict outcomes in either cohort [133]. These opposing results were concealed by the differences in treatment. The PRIMA trial regimen included doxorubicin (inducer of immunogenic cell death) and R maintenance that may benefit from high macrophage numbers.

In an attempt to solve these discrepancies and integrate the microenvironment in the prognosis algorithms, the Lunenburg Lymphoma Biomarker Consortium confirmed in a homogeneously rituximab-chemotherapy-treated group of patients that lower percentages of CD8^+^ T cells, CD163^+^ M2 macrophage areas, EZH2 wild-type status, and gain of chromosome 18 in the initial tumor biopsy specimen were predictors of poor prognosis FL treated with R-CHOP while refuting the prognostic impact of various other markers [134]. These results validate those of Kridel et al., showing that a higher CD163^+^ pixel count or CD163^+^ area were independent predictors of prolonged PFS in patients treated with R-CHOP, while the CD68^+^ macrophages population did not have a significant impact by pixel count or area [134].

Lately, the CSF1 receptor tyrosine kinase (CSF-1R) has generated attention. The colony-stimulating factor-1 (CSF-1) binds this receptor through autophosphorylation of CSF-1R. Triggering this phosphorylation cascade increases gene transcription and protein translation and induces cytoskeletal remodeling by several signaling pathways, leading to the recruitment, survival, proliferation, and differentiation of monocytes into macrophages. Because CSF-1 regulates the survival, proliferation, and chemotaxis of macrophages and supports their activation, this factor is involved in the pathogenesis of several diseases [135]. CSF-1R protein expression could represent an important tool in the future study of some lymphomas. Martin-Moreno et al. analyzed the distribution of CSF-1R^+^ cells in FFPE samples from reactive lymphoid tissues and different lymphoma types, including FL. The results demonstrated that the CSF-1R^+^ cell population only partially overlapped with the M2-type macrophages detected by CD163 expression, in agreement with previous observations about monocyte differentiation, where the *CSF-1R* gene was not significantly differentially expressed between M1 versus M2 monocyte activation models [136]. In this regard, recent results from our laboratory in FL patients homogeneously treated with R-CHOP yielded an association between high CSF-1R expression (both follicular and Interfollicular) and histological grade or risk of transformation [22].

Finally, an additional interesting checkpoint is CD47, a “do not eat me” antiphagocytic signal that forms a signaling complex with signal-regulatory protein α (SIRPα), enabling the escape of cancer cells from macrophage-mediated phagocytosis and other phagocytes. Virtually all cancers overexpress CD47. A growing number of studies have demonstrated that inhibiting the CD47-SIRPα signaling pathway promotes the adaptive immune response and enhances the phagocytosis of tumor cells by macrophages [137]. In a recent work using FFPE LN biopsies from FL patients, researchers identified three subsets (CD14^+^SIRPα^hi^, CD14^−^SIRPα^low^, and CD14^−^SIRPα^neg^) of monocytes/macrophages (Mo/MΦ) that exhibited specific differentiation, migration, phagocytic or immunosuppressive properties. When using SIRPα-Fc to block the interaction between SIRPα and CD47, alone or in combination with rituximab, phagocytosis of tumor cells was differentially increased in the three Mo/MΦ subsets. Clinically, high numbers of CD14^+^SIRPα^hi^ cells significantly correlated with poor prognosis in FL patients. In contrast, while not statistically significant, the number of CD14^−^SIRPα^low^ (that stimulate rather than suppress T cells) was associated with a favorable prognosis [71].

Overall, this better characterization of the supportive interactions between FL and TAMs has opened new therapeutic avenues. Four main approaches can be highlighted:

#### 5.2.1. Blockade of DC-SIGN-Mediated BCR Activation

BTK and SYK inhibitors have been demonstrated to reduce the viability of FL cells in vitro [23,25]. However, the clinical benefit of SYKi or BTKi in FL as a single agent are limited [63,65], and better results have been obtained in combination with anti-CD20 antibodies [64].

#### 5.2.2. Blockade of the CSF-1/CSF-1R Pathway Axis Has Been Extensively Investigated in Tumor Models and Is Paradigmatic of the TAM–Cancer Cell Interaction

This strategy represents a selective approach to manipulate macrophages and is well described in solid tumors and, more recently, in hematologic malignancies as mantle cell lymphoma [138] or acute myeloid leukemia [139]. In FL, the inhibition of CSF-1R kinase activity with PLX-3397 (pexidartinib) preferentially affects M2 macrophage viability and induces their repolarization to M1 macrophages, disrupting FL–M2 positive crosstalk. In vivo, CSF1-R inhibition caused M2 reduction and repolarization towards M1 macrophages and antitumor effect cooperating with anti-CD20 rituximab [22].

#### 5.2.3. PI3Kγ Inhibitors 

Although much attention has been paid to PI3Kδ inhibitors, the PI3Kγ isoform, highly expressed in leukocytes, plays a major role in cell chemotaxis to inflammation sites. Accordingly, PI3Kγ inhibition stimulates p65/RelA phosphorylation, promoting a more prone M1 phenotype in macrophages [140]. In HL and peripheral T cell lymphoma, PI3Kγ inhibition resulted in a shift of TMAs from the immunosuppressive M2-like phenotype to the inflammatory M1-like phenotype [141,142]. These additional properties of PI3Kγ may explain the good clinical results of the dual PI3Kδγ inhibitor duvelisib, currently approved for CLL, SLL, and FL [143].

#### 5.2.4. Blockade of CD47

Anti-CD47 antibodies induce an antitumor T cell response by the cross-presentation of tumor antigens by phagocytes to T cells. Hu5F9-G4 (hereafter, 5F9) is a humanized, IgG4 isotype, CD47-blocking monoclonal antibody [72]. In preclinical xenograft models, 5F9 enabled the phagocytic elimination of FL. CD47 is widely expressed on normal cells; however, 5F9 selectively eliminated malignant cells and not normal cells. Phagocytosis is dependent on unmasking pro-phagocytic “eat me” signals that are expressed only on tumor cells and not on normal cells (with the exception of aging red cells). In this phase 1b study, combination therapy with 5F9 plus rituximab was associated with a good safety profile and produced responses in half the patients with R/R aggressive and indolent lymphomas. The mechanism of antitumor synergy with 5F9 and rituximab therapy depends largely on macrophage-mediated tumor killing through the blockade of the antiphagocytic CD47 signal by 5F9 combined. Although a reduction in NK cell-mediated antibody-dependent cellular cytotoxic effect is a mechanism of rituximab resistance, these clinical data suggest that 5F9 can restore rituximab sensitivity by means of macrophage-mediated, antibody-dependent cellular phagocytosis. A phase II trial is currently ongoing (ClinicalTrials.gov Identifier: NCT02953509). 

### 5.3. Tumor-Associated Neutrophils in FL

Few data are available regarding interactions between FL cells and neutrophils, which are key players in the innate immune system. Although neutrophils are traditionally considered in the context of their antibacterial functions, there is increased awareness that tumor-associated neutrophils (TANs) may be key mediators of malignant transformation, tumor progression, angiogenesis, and the modulation of antitumor immunity.

Although TANs do not seem to be implicated in the phagocytosis mechanism of rituximab activity [144], in vitro and in vivo studies, have demonstrated that TANs may compromise the cytotoxic effect of common chemotherapeutic agents used through CD11b/ICAM-1 interaction with CD44 of malignant B cells and inhibit FL B cell apoptosis. Interestingly, the expression levels of activation markers (CD11a, CD11b, CD18, CD32, and CD66b) in TANs are significantly increased when neutrophils are co-cultured with FL cells, suggesting that lymphoma cells influence their phenotype and function [145]. In accordance, a clinical study demonstrated a correlation increased between neutrophil counts and reduced response rate to therapy, pointing out that these innate immune cells could be pharmacologically targeted to enhance therapeutic responses [146]. Likewise, the neutrophil-to-lymphocyte ratio (NLR) has been evaluated as a possible prognostic factor, demonstrating that NLR at relapse is associated with post-progression survival (PPS) as a continuous variable, where PPS was defined as the time from progression or relapse to the date of death [125].

## 6. Concluding Remarks

Over the last decade, thanks to the contribution of high-content techniques, both at genomic and cellular levels, a picture of the FL microenvironment has become much better delineated, providing the basis for the application of precision medicine. However, the road ahead is not completely clear, and a number of important topics remain to be answered. On the one hand, despite being a disease that is addicted to epigenetic mutations, no magic bullet has yet been found, and specific epigenetic modulators are eagerly awaited. On the other hand, in a disease where the immune microenvironment plays such a fundamental role, immunotherapy, besides anti-CD20 antibodies, is still far from being a reality. In this regard, there is an urgent need to better characterize the immune profile in specific clinical scenarios (i.e., early versus late relapse versus histological transformation) and tailor immunotherapies accordingly.

## Figures and Tables

**Table 1:** Current therapies in clinical or preclinical status targeting FL-microenvironment crosstalk.

Drug Family	Target	Status ^1^	Combination	Clinical Responses		Ref ^4^
				ORR ^2^ (%)	PFS ^3^ (Months)	
**Epigenetic Regulators**						
BRD3308	HDAC3i	PC	Anti-PDL1	NA		[49]
Vorinostat	HDACi	C	None	47	15.6	[51]
			None	49	20	[52]
Abexinostat	HDACi	C	None	63	20.5	[53]
Tazemetostat	EZH2	C	None	*mutEZH2*: 69 *wtEZH2*: 35	*mutEZH2*: 13.8 *wtEZH2*: 11.1	[55]
**Metabolic Regulators**						
Temsirolimus	mTOR	C	None	53.8	12.7	[56]
			Bendamustine and rituximab	90	22	[57]
			Bortezomib	56	16.5	[58]
**B-Cell Receptor Inhibitors**						
Idelalisib	PI3Kδ	C	None	57	11	[59]
		PC	Venetoclax	NA		[60]
Copanlisib	PI3Kαδ	C	None	58.7	11.2	[61]
		C	Nivolumab	NCT03884998		NA
		C	Rituximab	NCT03789240		NA
Duvelisib	PI3Kγδ	C	None	42	9.5	[62]
		C	Venetoclax	NCT03534323		NA
		C	Nivolumab	NCT03892044		NA
Ibrutinib	BTK	C	None	37.5	14	[63]
		C	Rituximab	85	41.9	[64]
Fostamatinib	SYK	C	None	10	4.2	[65]
Entospletinib	SYK	PC	Obinutuzumab	NCT03010358		NA
Cerdulatinib	SYK/JAK	C	None	>50%	NA	[66]
**Immune Checkpoints Inhibitors**						
CAR-T	HVEM	PC	None	NA		[67]
Nivolumab	PD1	C	None	40	NR	[68]
Pidilizumab	PDL-1	C	Rituximab	66	18.8	[69]
Sym022	LAG-3	C	None	NCT03489369		NA
		C	Anti-PD1	NCT03311412		NA
Sym023	TIM-3	C	None	NCT03489343		NA
		C	Anti-PD1	NCT03311412		NA
**Immune Checkpoint Activators**						
Urelumab	CD317	C	Rituximab	21	4.5	[70]
Selicrelumab	CD40	C	Anti-PD-L1	NCT03892525		NA
**Macrophage Checkpoint Inhibitors**						
Pexidartinib	CSF1-R	PC	Rituximab	NA		[22]
SIRPα-Fc	SIRPα	PC	Rituximab	NA		[71]
Hu5F9-G4 (5F9)	CD47	C	Rituximab	71	NR	[72]

^1^ C, Clinical, PC, Preclinical; ^2^ ORR, overall response rate; ^3^ PFS, progression free survival; ^4^ Ref, reference; NA, not available; NR, not reached.

## Data Availability

Not applicable.
